# Early warning of climate tipping points from critical slowing down: comparing methods to improve robustness

**DOI:** 10.1098/rsta.2011.0304

**Published:** 2012-03-13

**Authors:** T. M. Lenton, V. N. Livina, V. Dakos, E. H. Van Nes, M. Scheffer

**Affiliations:** 1 College of Life and Environmental Sciences, University of Exeter, Exeter EX4 4PS, UK; 2 School of Environmental Sciences, University of East Anglia, Norwich NR4 7TJ, UK; 3 Aquatic Ecology and Water Quality Management, Wageningen University, Wageningen, The Netherlands

**Keywords:** climate change, tipping point, bifurcation, early warning, deglaciation, thermohaline circulation

## Abstract

We address whether robust early warning signals can, in principle, be provided before a climate tipping point is reached, focusing on methods that seek to detect critical slowing down as a precursor of bifurcation. As a test bed, six previously analysed datasets are reconsidered, three palaeoclimate records approaching abrupt transitions at the end of the last ice age and three models of varying complexity forced through a collapse of the Atlantic thermohaline circulation. Approaches based on examining the lag-1 autocorrelation function or on detrended fluctuation analysis are applied together and compared. The effects of aggregating the data, detrending method, sliding window length and filtering bandwidth are examined. Robust indicators of critical slowing down are found prior to the abrupt warming event at the end of the Younger Dryas, but the indicators are less clear prior to the Bølling-Allerød warming, or glacial termination in Antarctica. Early warnings of thermohaline circulation collapse can be masked by inter-annual variability driven by atmospheric dynamics. However, rapidly decaying modes can be successfully filtered out by using a long bandwidth or by aggregating data. The two methods have complementary strengths and weaknesses and we recommend applying them together to improve the robustness of early warnings.

## 1. Introduction

An important question in climate forecasting is whether any early warning of an approaching threshold change or ‘tipping point’ in the climate system can be provided, before it is reached. In previous work, a suite of tipping elements in the climate system have been identified that may pass a tipping point under human-induced climate change [1]. Recent, somewhat abrupt climate changes add to the collective concern that larger future nonlinear changes pose a significant risk to societies [2]. Furthermore, recent assessments place such ‘large-scale discontinuities’ rather closer to the present state of the climate [3]. By definition, such events imply significant impacts on societies or on other living components of the Earth system. Hence, if an early warning of a climate tipping point can be achieved, then it could be of considerable value to societies, at least in helping them build an adaptive capacity to cope with what is approaching.

In general, for a system approaching a threshold where its current state becomes unstable, and it makes a transition to some other state, one can expect to see it become more sluggish in its response to small perturbations [4]. Mathematically speaking, for systems that can be characterized as gradually approaching a (co-dimension 1) bifurcation point in their equilibrium solutions, their leading eigenvalue tends towards zero, indicating a tendency towards infinitely slow recovery from perturbations. This is referred to as ‘critical slowing down’ in dynamical systems theory. This phenomenon has long been known about [5,6], but it has only recently been applied to climate dynamics [7,8].

Several approaches have been suggested for extracting the signal of critical slowing down from time-series data, by examining changes in spectral properties [8], autocorrelation [7,9] or memory [10] in the data. However, as yet, there has been only limited comparison of early warning methods being applied by different groups [11,12], and some uncertainty over their sensitivity to parameter choices used in the statistical analyses. This raises the problem of ‘false alarms’ (false-positives), which may arise because signals interpreted as indicative of approaching bifurcation are not statistically robust, or have other causes [13].

Here, we try to improve the robustness of early warning methods, by comparing two different approaches to detecting critical slowing down, previously applied independently by subsets of the authors [9,10]. Their sensitivity to data aggregation, methods of data detrending, window length and filtering bandwidth are examined. We also use two different testing grounds for the proposed early warning methods. The first is in palaeodata, where abrupt climate changes are seen to have occurred, and one considers the data leading up to the transition [9,10]. The second is in climate model simulations, where a model system is forced (usually very gradually) past a known threshold [7,9,10,14]. Here, we concentrate on six time series that we have previously analysed independently, three from each testing ground. In the models, we concentrate on a collapse of the Atlantic thermohaline circulation as the archetypal example of a climate tipping point involving bifurcation-type behaviour, based on models of varying complexity [14–17].

We note at the outset that several other potential indicators of approaching thresholds have been discussed and applied to ecological systems in the recent literature [11,12]. These include increasing variance [18], skewness [19], spatial correlation [20], spatial variance [21,22] and spatial skewness [22]. Potentially, the most robust early warning indicator will be some combination of different statistical properties of the data [13]. A combined indicator has been used to provide early warning of extinction in laboratory populations [12], but the particular ad hoc combination used was determined after the fact (i.e. once it was known that transitions had occurred). Ideally, we want a theoretically grounded *a priori* early warning indicator that is not case-specific. Motivated by these considerations, we also examine changes in variance in our chosen datasets below.

A further problem for any would-be tipping point early warning system is that of ‘missed alarms’ (false-negatives). This arises because not all candidate tipping points can be characterized by underlying bifurcations [1]. Also, abrupt noise-induced transitions can potentially occur in the climate system without any change in the stability properties of the initial climate state (i.e. the shape of the underlying potential) [13]. Such events are not expected to show any trend of critical slowing down prior to a transition (in contrast to slow forcing towards a bifurcation point). Current theory regarding abrupt climate changes in the palaeoclimate record suggests that the Dansgaard–Oeschger (DO) events during the last ice age can be characterized as noise-induced transitions [13,23]. However, there is some disagreement over whether the warming at the start of the Bølling-Allerød (BA; DO event 1) was preceded by slowing down [9] or not [13]. Below, we re-examine the robustness of this and two other candidates for slowing down in the palaeorecord.

Clearly, we cannot eliminate the possibility of ‘missed alarms’. Under future climate change, conceivably both bifurcations and noise-induced transitions could occur. Indeed, for systems subject to noise that are approaching a bifurcation point, they are likely to exit their initial state before the bifurcation point is reached. However, the methods we pursue here, based on diagnosing the slowest decay rate in a system, can provide some indicator of stability of the present state, and when combined with a diagnosis of the noise level, can give some indication of the vulnerability of a system to noise-induced transitions [24].

## 2. Methods

**Figure 1.**
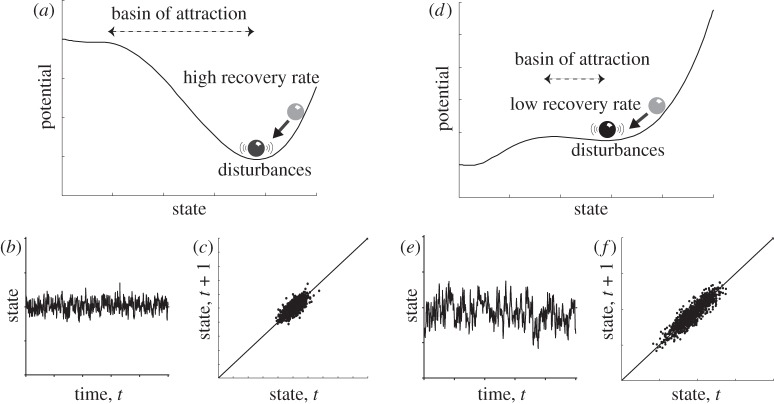
Heuristic illustration of critical slowing down. Panels show characteristic changes in non-equilibrium dynamics as a system approaches a tipping point (catastrophic bifurcation). (*a*–*c*) Far from the tipping point (*a*), the basin of attraction is steep and the rate of recovery from perturbations is relatively high, such that if the system is stochastically forced (*b*), the resulting dynamics are characterized by (*c*) low correlation between the states at subsequent time intervals and low variance. (*d*–*f*) When the system is closer to the tipping point (*d*), the basin of attraction shallows and the rate of recovery from small perturbations is lower, such that if the system is stochastically forced (*e*), the resulting dynamics are characterized by (*f*) a stronger correlation between subsequent states and a larger variance. (Adapted from Scheffer *et al*. [4].)

The methods compared here are based on the common principle of looking for critical slowing down in the dynamical response of a system as a bifurcation point is approached. Slowing down causes the intrinsic rates of change in a system to decrease, and therefore the state of the system at any given moment should become more and more like its past state, i.e. autocorrelation increases (figure 1). This increase in ‘memory’ can be measured in a variety of ways from the frequency spectrum of the system. Here, we concentrate on two approaches to extracting the signal of slowing down from data using the autocorrelation function (ACF), or detrended fluctuation analysis (DFA).

### (a) Autocorrelation function

Slowing down can be measured in its simplest form by an increase in lag-1 autocorrelation (how similar each point is to the previous one; figure 1). This is estimated by fitting an autoregressive model of order 1 (linear AR(1)-process) of the form
2.1
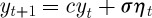

using an ordinary least-squares fitting method, where *η*_*t*_ is a Gaussian white noise process of variance *σ*^2^ and *c* is the autoregressive coefficient:
2.2
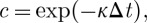

where *κ* is the decay rate of perturbations. If one aggregates the data to non-intersecting windows of length Δ*t*, such that 1/*κ*≫Δ*t*≫1/*κ*_*i*_, where *κ*_*i*_ is the decay rate of minor modes (not of interest), then one can directly extract the decay rate of the major mode, *κ*, which tends to zero (i.e. *c*→1) as bifurcation is approached [7]. The changing estimated value of the AR1 coefficient, *c*, as one moves through a time series is referred to here as the ‘ACF indicator’ (previously termed the ‘propagator’ [7]).

Somewhat surprisingly, even without any effort to aggregate data, increases in AR1 coefficient have been found in diverse time series approaching transitions [9] (perhaps because in the case of palaeorecords they already tend to integrate over long time scales). The absolute value of the AR1 coefficient reflects the ratio between the time scale of the dynamics and the frequency of measurements. Thus, high values can simply reflect regular measurements relative to the frequency of internal fluctuations. As a consequence, we use an upward trend in the ACF indicator as the primary early warning signal, rather than its absolute value. The indicator value and trend are also sensitive to, for example, data detrending and measurement noise. Also, in a system with stochastic forcing, the larger the amplitude of that forcing, the further from bifurcation a transition is expected to occur [24].

### (b) Detrended fluctuation analysis

Slowing down causes an increase in short-term memory (figure 1), which can also be measured using DFA. DFA extracts the fluctuation function of window size *s*, which increases as a power law if the data series is long-term power law-correlated:
2.3
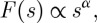

where *α* is the DFA scaling exponent. We consider only the short-term regime, in which as *c*→1 and the data approach critical behaviour, the slowing exponential decay is well approximated by a power law in which *α*→1.5 (corresponding to a random walk). The DFA exponent is rescaled to give a ‘DFA indicator’ that has been calibrated against the ACF indicator for direct comparison, and reaches value 1 (rescaled from 1.5) at critical behaviour [10]. Again, we use an upward trend in the DFA indicator as the primary early warning signal, rather than its absolute value.

### (c) Data processing

Data can be aggregated prior to analysis by simply averaging over non-intersecting windows of chosen length. Owing to possible non-stationarities in the palaeoclimate and simulated records, it is useful to remove trends before estimating the slowing-down indicators. The DFA method includes an inherent, internal detrending routine, described in detail elsewhere [10]. Here, it is of low-order and equivalent to simple linear detrending. For the ACF approach, we have explored several different approaches. Firstly, we estimated the ACF indicator without detrending the data as in Lenton *et al*. [14]. Then we focused on the following two detrending methods.

— *Linear detrending* is the simplest technique to cope with non-stationarities in mean value in time series as used in Held & Kleinen [7]. Before calculating lag-1 autocorrelations, we subtract the linear trend in each window and thus obtain a locally quasi-stationary time series. This detrending makes sense only when applied in windows of small or medium size, because in large windows, simple linear detrending of highly non-stationary data introduces large bias. Comparing the resulting indicator with that of the initial data (without detrending) provides information about the presence of any trend in the data, and the particular time interval where this trend affects the data.
— *Gaussian filtering* fits a Gaussian kernel smoothing function across the whole record prior to transition as used in Dakos *et al*. [9]. The fit is subtracted from the record to obtain the residual data series. The choice of bandwidth for the kernel determines the degree of smoothing. As a rule of thumb, a bandwidth is chosen in such a way that it neither over-fits the data nor filters out low frequencies in the record. We also tested the performance of the ACF indicator when Gaussian filtering was applied only within the sliding window.

ACF and DFA early warning indicators are estimated within a sliding window over a time series preceding the onset of a transition. The choice of the sliding window length is a trade-off between time-resolution (data availability) and reliability of the estimate for the indicators. Here, instead of using a default value, sensitivity analyses were performed where the length of the sliding window was varied from 25 per cent of the record length up to 75 per cent using increments of 20 points. In the case of the ACF indicator, the bandwidth size of the Gaussian filtering was also varied [9]. Estimates of the indicators from these sensitivity analyses were used to identify trends before the transition. Trends were quantified using the non-parametric Kendall *τ* rank correlation coefficient [25]. A positive Kendall *τ* coefficient is a measure of increasing trends in the indicators prior to transition.

### (d) Data sources

We cut off each time series before the start of a distinct transition in the data. Here, we examine three palaeodata records all showing aspects of the end of the last ice age, but covering different time frames and coming from different regions.

— *Vostok*. The Vostok ice core deuterium record [26] is a proxy for local temperature in Antarctica. We consider the interval from 58 800 years BP until 17 000 years BP (*n*=513 points, interpolated data) as in Dakos *et al*. [9], which includes several Antarctic cold-reversal events and the last glacial maximum, but excludes the deglaciation known as ‘termination I’.
— *GISP2*. The GISP2 ice core *δ*^18^O record [27] is a proxy for local temperature in central Greenland. We consider the interval 21 000–14 800 years BP (*n*=132 points, interpolated data) as in Dakos *et al*. [9], which begins after the interval of pronounced DO events, and stops just before the transition into the BA warm interval.
— *Cariaco*. The Cariaco basin sediment core PL07-58PC greyscale record [28] is a high-resolution proxy for local productivity in the tropical Atlantic, which is known to correlate with climate changes recorded in Greenland. We consider the interval from 12 500 to 11 600 years BP (*n*=2111 points, interpolated data) as in [9], which spans the Younger Dryas, but excludes the abrupt drop in productivity at its end.

We also examine the time series from three model experiments, each involving a gradually forced collapse of the Atlantic thermohaline circulation. Our three chosen models span a range of intermediate complexity.

— *CLIMBER-2* has a zonally averaged (two-dimensional) model for each ocean basin, connected by an Antarctic circumpolar current, and coupled to a statistical dynamical atmosphere model. White noise is applied in order to mimic short-time-scale variability driven by the atmosphere. The model is forced with a linear increase in atmospheric CO_2_ from 280 to 800 ppm over 50 000 years, driving increased freshwater forcing at 44^°^ N, and forcing a collapse of the thermohaline circulation after about 39 000 years. The model output is aggregated to Δ*t*=50 year time steps (*n*=783) and is the first principal component of salinity as in Dakos *et al*. [9] and Held & Kleinen [7].
— *GENIE-1* has a three-dimensional frictional geostrophic ocean, coupled to an energy and moisture balance atmosphere model, and dynamic sea-ice. White noise is applied in order to mimic short-time-scale variability driven by the atmosphere. The model is forced with a 0.003% yr^−1^ increase in CO_2_ from 280 ppm that forces a collapse of the thermohaline circulation after about 38 000 years. The annual output analysed (*n*=37 600) is the maximum Atlantic meridional overturning circulation below 500 m as in Livina & Lenton [10].
— *GENIE-2* has a full primitive equation, three-dimensional, dynamical atmosphere coupled to the same three-dimensional ocean model as GENIE-1 and slab sea-ice. The dynamical atmosphere drives inter-annual variability in the ocean circulation. The model is forced with a gradual 0.05 Sv kyr^−1^ increase in freshwater forcing in the region 50–70^°^ N, which forces a collapse of the thermohaline circulation after about 5300 years. The annual output analysed (*n*=5270) is the maximum Atlantic meridional overturning circulation below 500 m as in Lenton *et al*. [14]. We also analysed aggregated data averaged to Δ*t*=5 years (*n*=1054), 10 years (*n*=527) or 20 years (*n*=263) resolution.

**Figure 2.**
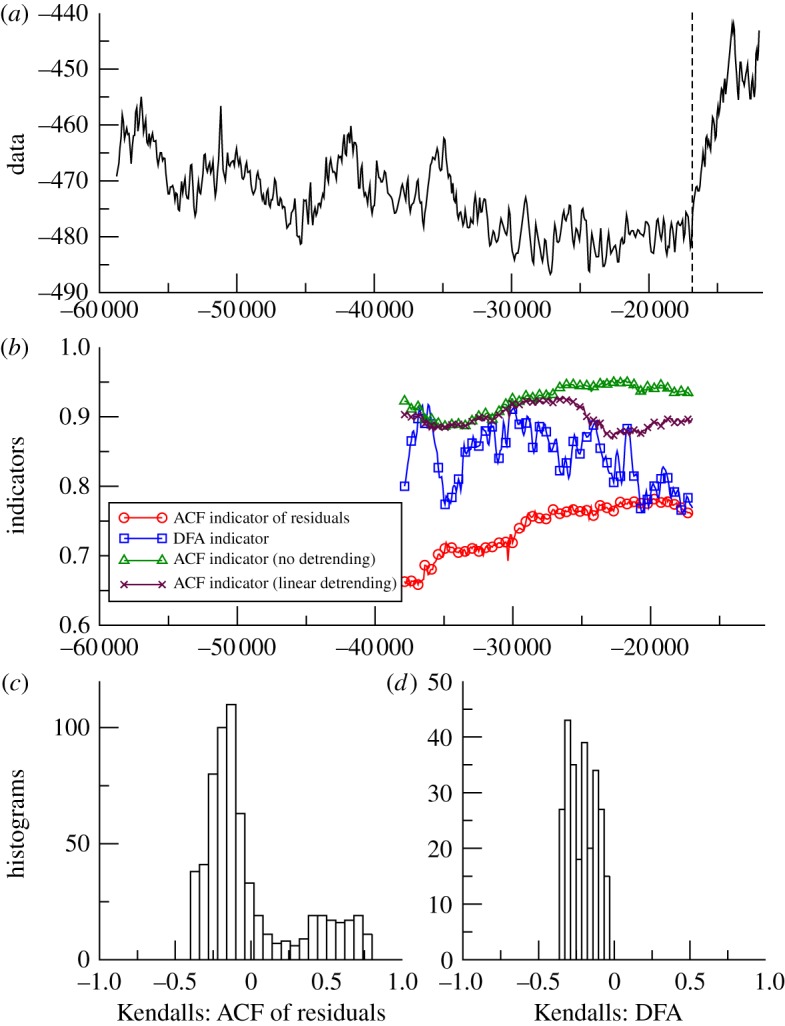
Search for early warning of the last deglaciation as seen in Antarctica. (*a*) Vostok deuterium proxy for local temperature 58.8–17 kyr BP (*n*=513). Analysis stops at the vertical dashed line before the termination occurs. (*b*) Example of early warning indicators from autocorrelation function (ACF) with various detrending methods, or detrended fluctuation analysis (DFA); results plotted at the end of the windows (sliding window length of half the data series in all cases, bandwidth = 25 for ACF method). (*c*) Histogram of the frequency distribution of the Kendall trend statistic for the ACF of residuals indicator, when varying the sliding window length and filtering bandwidth. (*d*) Histogram of the frequency distribution of the Kendall trend statistic for the DFA indicator, when varying the sliding window length. (Online version in colour.)

The results for these six datasets are plotted in a common format with: (figure 2*a*) time series; (figure 2*b*) example of ACF and DFA indicators (with a sliding window length of half the data series, results plotted at the end of the windows); (figure 2*c*) histogram of the frequency distribution of the trend statistic for the ACF indicator, when varying the sliding window length and filtering bandwidth; and (figure 2*d*) histogram of the frequency distribution of the trend statistic for the DFA indicator, when only varying the sliding window length.

## 3. Results

First, we consider the analyses of palaeodata approaching abrupt transitions, where we are unsure whether there is an underlying bifurcation.

*Vostok*. Termination I (the end of the last ice age) as recorded in Antarctica is preceded by an upward trend in the ACF indicator (figure 2*b*), when using the original Gaussian detrending method and parameter choices (sliding window of half the series length, filtering bandwidth of 25). Linear detrending, contrasted with no detrending, suggests that the trend in the data affects the latter part of the results, and the more sophisticated Gaussian detrending is able to cope better with non-stationarity in the data. The positive trend in the ACF indicator is robust for the examined range of window lengths and for filtering bandwidth ≤70, but longer bandwidths give negative Kendall statistics (i.e. decreasing trends; figure 2*c*). Such long bandwidths can be ruled out, because visual inspection shows that the kernel width becomes too large to follow the trend in the data [9]. However, the DFA indicator gives a slightly negative trend when using a sliding window of half the series length (figure 2*b*), and the Kendall statistics are robustly negative for a range of window lengths (figure 2*d*).

**Figure 3.**
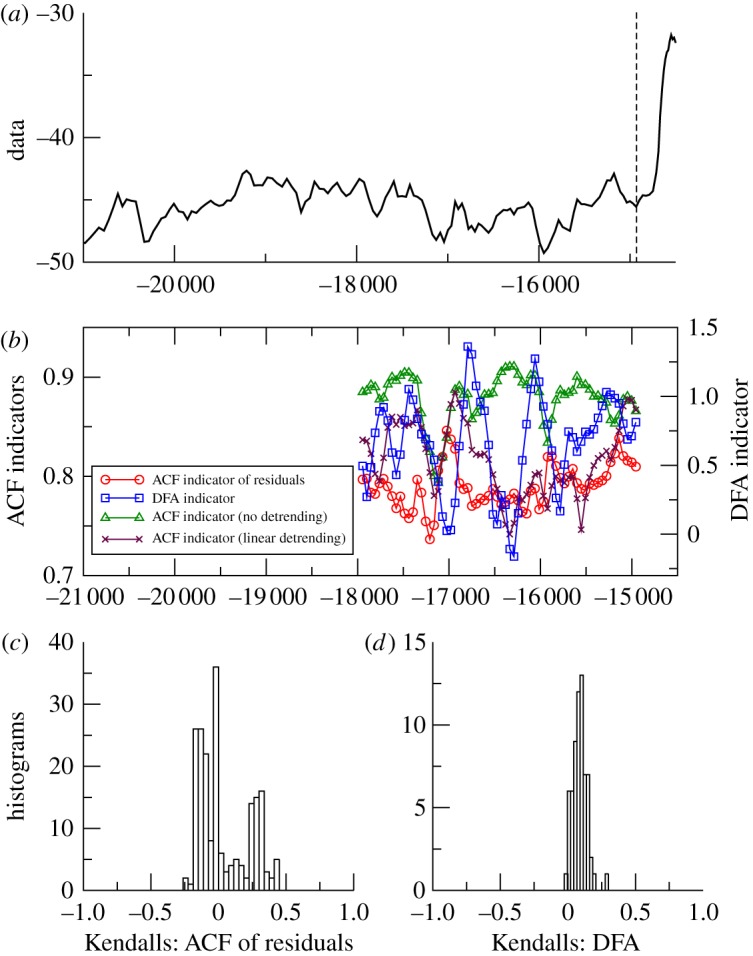
Search for early warning of the Bølling-Allerød transition as seen in Greenland. (*a*) GISP2 *δ*^18^O proxy for local temperature, 21.0–14.8 kyr BP (*n*= 132) [9]. Analysis stops at the vertical dashed line before the transition into the Bølling-Allerød warm interval. (*b*) Example of early warning indicators from autocorrelation function (ACF) with various detrending methods (left-hand scale), or detrended fluctuation analysis (DFA, right-hand scale); results plotted at the end of the windows (sliding window length of half the data series in all cases, bandwidth=25 for ACF method). (*c*) Histogram of the frequency distribution of the Kendall trend statistic for the ACF of residuals indicator, when varying the sliding window length and filtering bandwidth. (*d*) Histogram of the frequency distribution of the Kendall trend statistic for the DFA indicator, when varying the sliding window length. (Online version in colour.)

*GISP2*. The warming marking the start of the BA interval is preceded by an overall upward trend in the example of ACF indicator based on residuals, but the upward trend in the example of DFA indicator is obscured by large fluctuations (figure 3*b*). These are to be expected with such a short time series. The ACF indicator gives either positive or negative trends depending on the choice of sliding window length and filtering bandwidth (figure 3*c*). Negative trends are found for either very short bandwidth (<10) or a combination of long bandwidth (>50) and window length half the series or greater. However, the positive trend in the DFA indicator is robust (if small) for a range of window lengths (figure 3*d*).

**Figure 4.**
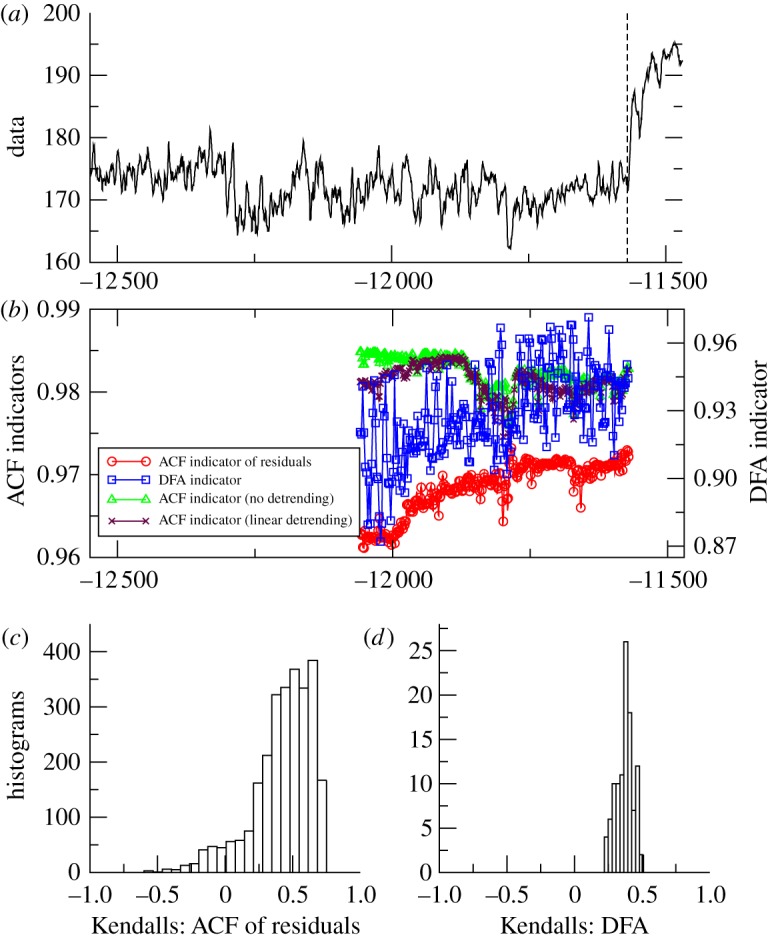
Search for early warning of the end of the Younger Dryas in the tropical Atlantic. (*a*) Cariaco basin core PL07-58PC greyscale proxy for local productivity, 12.5–11.6 kyr BP (*n*=2111). Analysis stops at the vertical dashed line before the transition into the Holocene. (*b*) Example of early warning indicators from autocorrelation function (ACF) with various detrending methods (left-hand scale), or detrended fluctuation analysis (DFA, right-hand scale); results plotted at the end of the windows (sliding window length of half the data series in all cases, bandwidth = 100 for ACF method). (*c*) Histogram of the frequency distribution of the Kendall trend statistic for the ACF of residuals indicator, when varying the sliding window length and filtering bandwidth. (*d*) Histogram of the frequency distribution of the Kendall trend statistic for the DFA indicator, when varying the sliding window length. (Online version in colour.)

*Cariaco*. The end of the Younger Dryas, as recorded in the Cariaco basin, is preceded by an overall upward trend in the example of ACF and DFA indicators (figure 4*b*). There are again distinct fluctuations in the DFA indicator. Gaussian detrending is necessary to cope with non-stationarity in the data when applying the ACF method. The positive trend in the resulting ACF indicator is robust across most window lengths and bandwidths (figure 4*c*). Only a combination of short filtering bandwidth and long sliding window picks up negative trends. The positive trend in the DFA indicator is robust across all window lengths (figure 4*d*).

We now turn to the model results for collapse of the thermohaline circulation, where we know that the systems are approaching an underlying bifurcation.

**Figure 5.**
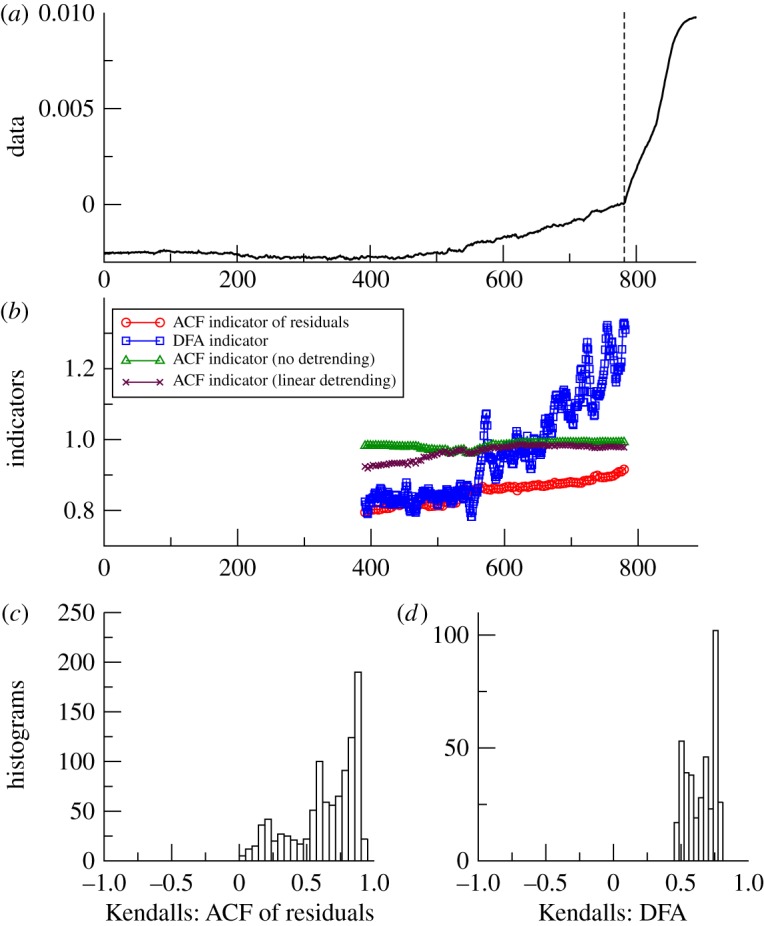
Search for early warning of Atlantic thermohaline circulation collapse in the CLIMBER-2 model. (*a*) Principal component of salinity, as freshwater forcing is gradually increased and white noise is applied in order to diagnose ocean dynamics. Analysis stops at the vertical dashed line before the transition (*n*=783). (*b*) Example of early warning indicators from detrended fluctuation analysis (DFA) or autocorrelation function (ACF) with various detrending methods and results plotted at the end of the windows (sliding window length of half the data series in all cases, bandwidth=50 for ACF method). (*c*) Histogram of the frequency distribution of the Kendall trend statistic for the ACF of residuals indicator, when varying the sliding window length and filtering bandwidth. (*d*) Histogram of the frequency distribution of the Kendall trend statistic for the DFA indicator, when varying the sliding window length. (Online version in colour.)

*CLIMBER-2*. Thermohaline circulation collapse in CLIMBER-2 shows an overall upward trend in the example of ACF and DFA indicators, with some fluctuations (figure 5*b*). Linear detrending also produces an upward trend in the example and shows that detrending particularly affects the first part of the results. The positive trend in the ACF indicator is robust across all window lengths and bandwidths considered (figure 5*c*), consistent with previous results [7,9]. The positive trend in the DFA indicator is robust across all window lengths (figure 5*d*). (The example of DFA indicator exceeds the critical value of 1 prior to the transition, thus providing a ‘too early’ warning, if one were to concentrate on its absolute value.)

**Figure 6.**
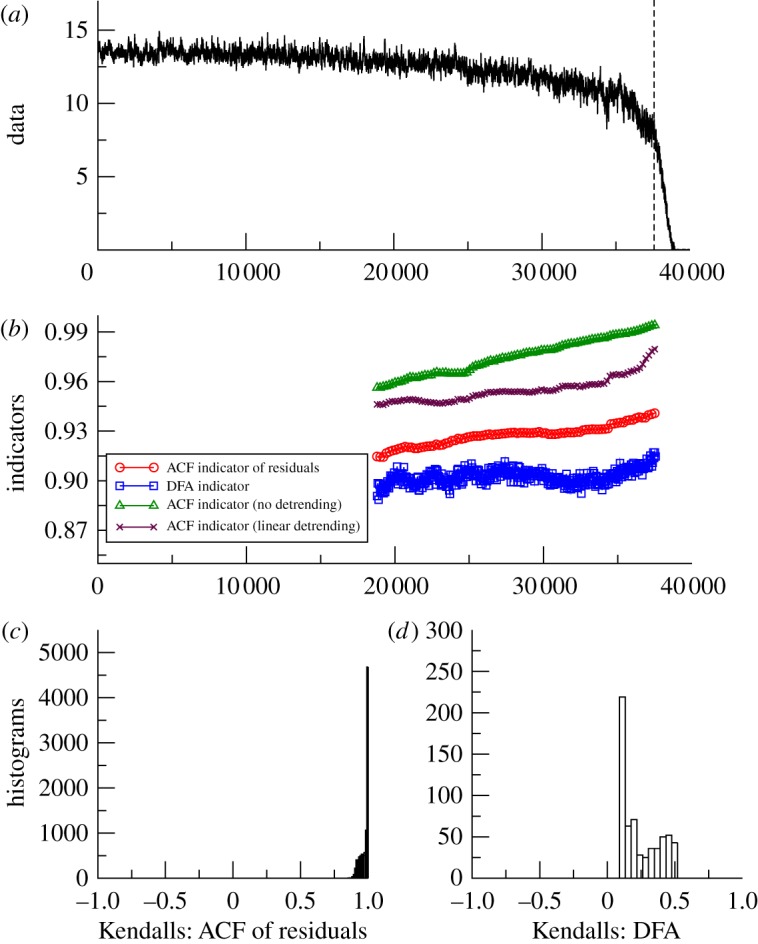
Search for early warning of Atlantic thermohaline circulation collapse in the GENIE-1 model. (*a*) Strength of the Atlantic meridional overturning circulation (Sv) as freshwater forcing is gradually increased, and white noise is applied in order to diagnose ocean dynamics. Analysis stops at the vertical dashed line before the transition (*n*=37 600). (*b*) Example of early warning indicators from detrended fluctuation analysis (DFA) or autocorrelation function (ACF) with various detrending methods and results plotted at the end of the windows (sliding window length of half the data series in all cases, bandwidth=100 for ACF method). (*c*) Histogram of the frequency distribution of the Kendall trend statistic for the ACF of residuals indicator, when varying the sliding window length and filtering bandwidth. (*d*) Histogram of the frequency distribution of the Kendall trend statistic for the DFA indicator, when varying the sliding window length. (Online version in colour.)

*GENIE-1*. Thermohaline collapse in GENIE-1 shows an overall upward trend in the example of ACF and DFA indicators (figure 6*b*). Even without detrending (or with linear detrending) there is a distinct upward trend. The positive trend in the ACF indicator is extremely robust across all window lengths and bandwidths considered (figure 6*c*). The positive trend in the DFA indicator is also extremely robust (albeit smaller) across all window lengths (figure 6*d*), consistent with previous results (using a shorter window of 5000 data points or 13.5% of the series) [10].

**Figure 7.**
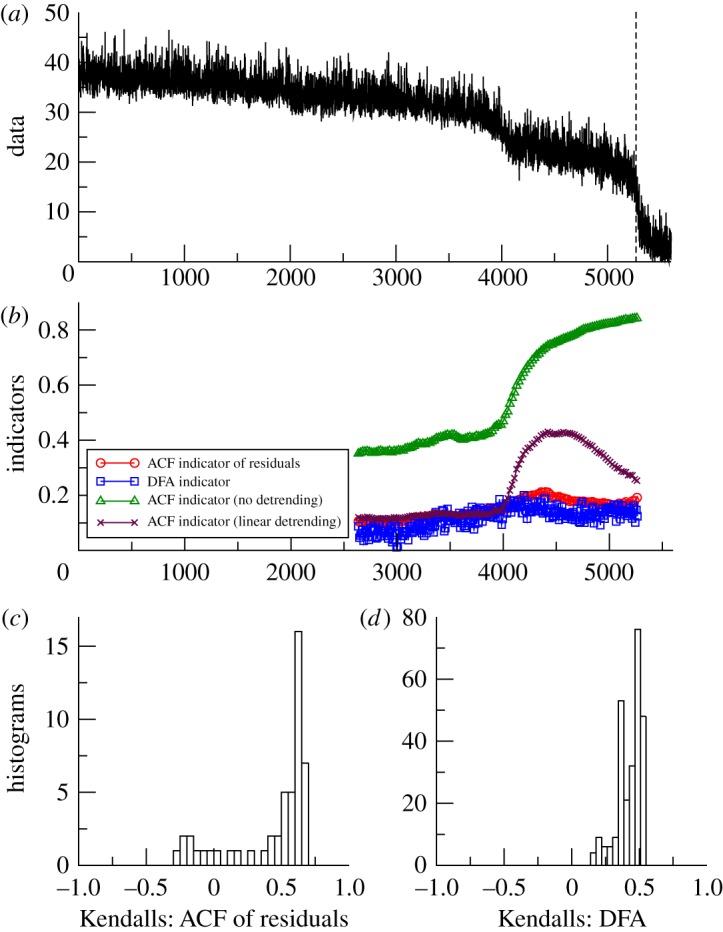
Search for early warning of Atlantic thermohaline circulation collapse in the GENIE-2 model. (*a*) Strength of the Atlantic meridional overturning circulation (Sv) as freshwater forcing is gradually increased, with the dynamical atmosphere driving short-term variability in the ocean circulation. Analysis stops at the vertical dashed line before the transition (*n*=5270). (*b*) Example of early warning indicators from detrended fluctuation analysis (DFA) or autocorrelation function (ACF) with various detrending methods and results plotted at the end of the windows (sliding window length of half the data series in all cases, bandwidth=1000 for ACF method). (*c*) Histogram of the frequency distribution of the Kendall trend statistic for the ACF of residuals indicator, when varying the sliding window length and filtering bandwidth. (*d*) Histogram of the frequency distribution of the Kendall trend statistic for the DFA indicator, when varying the sliding window length. (Online version in colour.)

**Figure 8.**
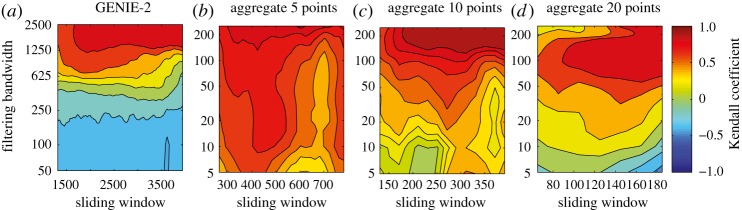
The effect of data aggregation on early warning of thermohaline circulation collapse in a coupled ocean–atmosphere model. Based on aggregating results from the GENIE-2 model (figure 7*a*). Contour plots show the value of the Kendall trend statistic for the ACF indicator derived after detrending by Gaussian filtering, when varying the sliding window length and filtering bandwidth. Analysis based on (*a*) raw data (as in figure 7*c*), (*b*) data averaged to Δ*t*=5 years (*n*=1054), (*c*) Δ*t*=10 years (*n*=527) or (*d*) Δ*t*=20 years (*n*=263) resolution. Note the change in bandwidth range between (*a*) and (*b*–*d*).

*GENIE-2*. Thermohaline circulation collapse in GENIE-2 shows conflicting trends in the indicators for the annual data of circulation strength (figure 7). Without detrending, or with linear detrending, the ACF indicators rise markedly associated with the transition in the data around year 4000 [14], suggesting that more sophisticated detrending is required. The DFA indicator also shows a positive trend in the example (figure 7*b*) [14], which is robust when varying the window length (figure 7*d*). After applying Gaussian filtering, there are mixed trends in the ACF indicator (figure 7*c*). Only for a filtering bandwidth exceeding about one-tenth of the length of the series are there positive trends, which are strongest for a bandwidth of around half of the series (figure 8*a*). (The example indicators are far from the critical value of 1 when transition occurs, but that is to be expected in a system with such a high level of internal variability.)

The GENIE-2 results (figure 7*c*) suggest that with the raw data and relatively short bandwidths, we may be sampling the decay of fast modes in a system, which are not relevant to detecting approaching bifurcation. We think that these fast modes reflect the dynamics of coupling to the overlying atmosphere model, which produces strong inter-annual variability in the strength of the thermohaline circulation. Hence, we tried aggregating the GENIE-2 output over longer time scales in order to try and reveal the underlying ocean dynamics. This has a profound effect on the results (figure 8). On aggregating the results to Δ*t*=5 year resolution, the trend in the ACF indicator becomes robustly positive, with some slight degradation on aggregating further to 10 or 20 year resolution (although of course, data are being lost on increasing the aggregation).

The effects of detrending within sliding windows as opposed to across the whole series are relatively minor in all our datasets (electronic supplementary material, figure A1).

**Figure 9.**
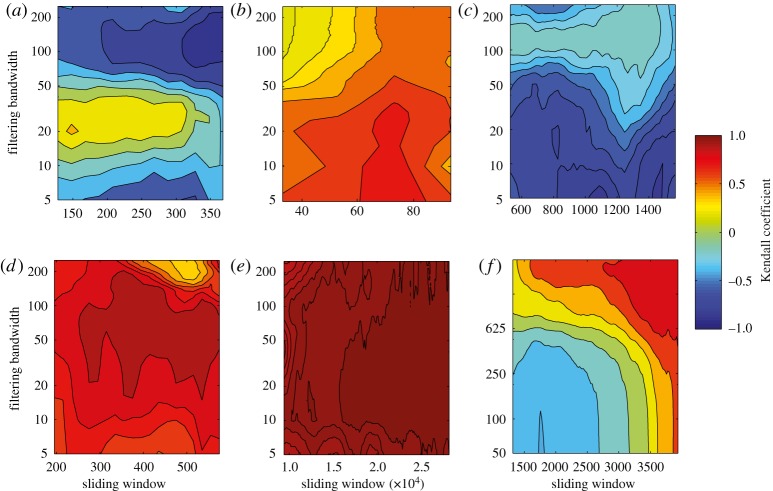
Trends in variance as a threshold is approached for all the datasets. Contour plots show the value of the Kendall trend statistic for the trend in standard deviation, derived after detrending by Gaussian filtering across the whole series, when varying the sliding window length and filtering bandwidth. For different datasets: (*a*) Vostok, (*b*) GISP2, (*c*) Cariaco, (*d*) CLIMBER-2, (*e*) GENIE-1, (*f*) GENIE-2 (raw data).

Finally, trends in variance differ between the datasets as they approach abrupt transitions (figure 9). Upward trends in variance are robust for the GISP2 record, CLIMBER-2 and GENIE-1. However, there are mixed trends in variance in GENIE-2 (without data aggregation), predominantly downward trends in the Vostok record, and universally downward trends in the Cariaco record.

Table 1 summarizes the results in terms of which early warning indicator worked for which dataset.

**Table 1.** Summary of which early warning indicator worked for which dataset. Symbols indicate the sign(s), and numerical values the mean ± s.d. of the Kendall trend statistics obtained when varying the sliding window length and (for ACF and variance) the filtering bandwidth.

	early warning signal?					
dataset	ACF		DFA		variance	
Vostok	−/+	0±0.32	−	−0.21±0.09	−(+)	−0.48±0.31
GISP2	+(−)	0.04±0.18	+	0.09±0.05	+	0.45±0.17
Cariaco	+(−)	0.42±0.22	+	0.37±0.06	−	−0.31±0.16
CLIMBER-2	+	0.64±0.24	+	0.65±0.10	+	0.79±0.16
GENIE-1	+	0.97±0.03	+	0.25±0.14	+	0.98±0.03
GENIE-2	+(−)	0.58±0.21	+	0.43±0.09	−(+)	−0.07±0.31

## 4. Discussion

From the palaeodatasets analysed, approaching abrupt transitions, the results are mixed (table 1). They lead us to question whether there is slowing down prior to termination I in the Vostok data. The ACF method with justifiable filtering parameter choices suggests so [9], but the DFA method gives the opposite result (figure 2). Hence, more work is needed, ideally with higher resolution data, to resolve whether slowing down preceded the end of the last glaciation in Antarctica. Slowing down in the GISP2 data approaching the start of the BA is consistent across the two methods, but the trend is weak (table 1) and particular parametrizations within the ACF method suggest the opposite (figure 3). This is especially true for small sliding window size (electronic supplementary material, figure A1), which means that the GISP2 time series is too short for robust estimates of slowing down, and further work with higher resolution ice core data is needed. In contrast, in the Cariaco basin data approaching the end of the Younger Dryas, there is a robust signal of slowing down from both methods (table 1), which is also largely robust to varying the parameters within the methods (figure 4), reinforcing previous results [9].

From a mechanistic perspective, whether the onset of deglacial warming in Antarctica as recorded at Vostok should be characterized as a bifurcation is debatable. While Northern Hemisphere ice volume may exhibit multiple stable states and bifurcations [29], it lags the start of warming in Antarctica [30]. An argument could be made that positive feedbacks on global temperature change approached the runaway condition at the start of termination I. However, the start of deglaciation appears essentially indistinguishable from previous Antarctic warming events during the ice age [31]. As for the abrupt changes in climate during deglaciation, there are hypothesized links to transitions (if not bifurcations) in the Atlantic thermohaline circulation. For example, it has been hypothesized that meltwater pulse 1A originated from Antarctica causing abrupt strengthening of overturning and contributing to the BA warming [32] (a noise-induced transition rather than a bifurcation scenario). The abrupt transition at the end of the Younger Dryas, recorded in the Cariaco basin (and in many other locations), could also be linked to a change in the Atlantic thermohaline circulation. However, both are candidates for an abrupt strengthening rather than a collapse of the thermohaline circulation. Hence, our data and model tests should be viewed largely independently.

From the model experiments analysed, where we know that the systems are approaching an underlying bifurcation, both methods provide consistent early warning indicators in simpler models with white noise applied (table 1). Of the three models, the more complex GENIE-2 is the most instructive of what may happen in real-world applications of early warning indicators, because like the real world, the model includes coupled dynamical components with very different internal time scales, the atmosphere and ocean. Our analysis suggests that inter-annual variability in overturning strength in the model ocean primarily reflects coupling to the overlying atmosphere dynamics. Hence, analysing the original, annual resolution dataset with the ACF method and short bandwidth amounts to sampling rapid decay modes that are not pertinent to bifurcation detection (and which appears to be ‘speeding-up’ as the thermohaline circulation approaches collapse). However, consistent with the short memory of the atmosphere, using either a longer filtering bandwidth or aggregating the data to a 5-year time scale is sufficient to reveal underlying slowing down in the ocean dynamics. Much of the trend in the ACF indicator is in the lead up to distinct weakening of the overturning circulation (around year 4000 in figure 7), prior to the eventual collapse, suggesting that this transition be re-examined for hysteresis behaviour and possible underlying bifurcation [33].

We find mixed trends in variance across the different datasets (figure 9 and table 1), which can be rationalized. In the models of thermohaline circulation collapse, the systems approach a saddle-node bifurcation in which variance is expected to rise in proportion to autocorrelation (so they are not independent indicators) [13]. This shows up extremely clearly for GENIE-1 and CLIMBER-2, where the systems are forced very slowly and large datasets are available, although it is unclear in the GENIE-2 results. However, we do not expect rising variance to be a universal property of systems approaching bifurcation. When noise is acting on the processes of a system, variance may decrease as well as increase prior to a bifurcation [34]. Also, in the case of increasing memory before a bifurcation (when the data become equivalent to a random walk), the variance, at limit, becomes infinite, and therefore monitoring it in windows of fixed size may be misleading [35,36]. In the palaeodatasets approaching abrupt transitions, there are quite different trends in variance (figure 9). In the Vostok data, any trend is ambiguous. In the GISP2 data, there is a positive trend in variance, not seen in previous analyses of a higher resolution north Greenland ice core record [13]. In contrast, in the Cariaco basin data, the trend in variance is robustly negative, as can be seen by eye (figure 4*a*; the variance tends to rise and then fall on progressing through the dataset).

These trends in variance in palaeodata can potentially be linked to trends in noise level. The rise in variance in the GISP2 data might be attributable to deglaciation causing pulses of freshwater forcing that in turn drove increased fluctuations in the Atlantic thermohaline circulation and hence Greenland climate. Increased likelihood of a noise-induced transition can come with increased fluctuations, consistent with the hypothesis that the BA was basically noise-induced [13]. In contrast, the decline in variance during the Younger Dryas in the Cariaco basin data could reflect declining fluctuations in freshwater forcing of the Atlantic as Northern Hemisphere ice sheets re-stabilized during this cold interval. The fact that an abrupt transition still occurred at the end of the Younger Dryas, despite declining noise level, may strengthen the case for an underlying bifurcation. However, the effect of noise on the likelihood of transitions depends on whether noise is multiplied by, or added to, the state variable of a system [37].

Overall, the DFA method appears to give the least ambiguous results (table 1). However, this may simply reflect the fact that only the sliding window length is varied in the DFA method, whereas for the ACF method and the variance calculation, filtering bandwidth is also varied, and this tends to cause greater variation in the sign of the Kendall trend statistic.

## 5. Conclusion

From our results, we can offer some general guidelines for analyses aimed at detecting the signal of critical slowing down as an early warning of approaching bifurcation.

Prior to trying to extract early warning indicators, it is important to consider aggregating data where it may be of sufficiently high temporal resolution to be sampling fast decay modes in the system in question. This judgement should ideally be based on relevant mechanistic understanding. However, the possibility of fast decay modes dominating the signal seems less acute than might be expected. In the case of the ocean thermohaline circulation, Held & Kleinen [7] recommend Δ*t*=50 years to eliminate the fast modes, but Δ*t*=5 years appears sufficient in our GENIE-2 simulations.

Next, it is important to carefully detrend the data to remove any possible non-stationarities (detrending is inherent to the DFA method but not the ACF method). In the datasets we have considered, linear detrending is sometimes insufficient, but the more sophisticated Gaussian filtering works well. Whether detrending is conducted across the whole series or within a window makes little difference in the examples considered here.

Having obtained an appropriate time series, the two methods applied have different pros and cons (table 2). The ACF method is simpler, faster to calculate and more intuitive, but the indicator is bounded by an upper limit and therefore loses sensitivity near critical behaviour. The DFA indicator is more sensitive, providing extra information about the dynamics near critical behaviour, but is more data-demanding, oscillating when using short time series, thus making it less applicable to them. Also, the DFA method is more complex and slower to calculate. Overall, the methods complement one another, and we recommend applying both together as a cross-check.

**Table 2.** Summary of the pros and cons of the two methods for providing early warning indicators.

	ACF	DFA
data requirements	average	high
computational requirements	low	high
detrending	optional	built-in
parameter sensitivity	sliding window length, filtering bandwidth	sliding window length
slowing down estimate	underestimated (when applying detrending)	sensitive close to critical point
trend prior to bifurcation	smooth	oscillating

The choices of sliding window length (for both methods) and filtering bandwidth (ACF method) clearly influence the results. Short sliding windows give less reliable and more fluctuating indicators. Large sliding windows provide smoother estimates. A very narrow bandwidth filtering removes short-term fluctuations, and may completely eliminate the low frequencies we need for estimating slowing down. A wide bandwidth may fail to successfully remove the underlying trends in the data, and the remaining non-stationarities may cause spurious autocorrelation. A sensitivity analysis for both parameters is prudent, and the final choices should be guided by the resolution and length of the data, as well as known or observed trends in the dataset. Some theoretical guidelines for choosing the value of both parameters are given elsewhere [24]. For the thermohaline circulation experiments, theory would suggest both a bandwidth and window length of the order of a few thousand years would sit between the very slow time scale of forcing and the (initially) faster time scale of recovery of the critical mode. This is supported by the GENIE-2 results (figure 8).

Our results confirm that early warning signals can, in principle, be seen before a climate tipping point is reached. In particular, slowing down is detectable prior to abrupt transitions in model experiments, and in palaeodata prior to the end of the Younger Dryas. Previously detected slowing down prior to glacial termination in Antarctica does not appear robust. The robustness of the slowing down signal prior to the BA warming in Greenland deserves further examination, even though this was probably a noise-induced transition [13]. In a separate work, we are applying the early warning methods to higher resolution Greenland ice core data, to build up a more complete picture of changing climate stability through the last deglaciation.
